# Isotocin neuronal phenotypes differ among social systems in cichlid fishes

**DOI:** 10.1098/rsos.170350

**Published:** 2017-05-17

**Authors:** Adam R. Reddon, Constance M. O'Connor, Erin Nesjan, Jason Cameron, Jennifer K. Hellmann, Isaac Y. Ligocki, Susan E. Marsh-Rollo, Ian M. Hamilton, Douglas R. Wylie, Peter L. Hurd, Sigal Balshine

**Affiliations:** 1 Department of Biology, McGill University, Montreal, Quebec, Canada; 2 Department of Psychology, Neuroscience and Behaviour, McMaster University, Hamilton, Ontario, Canada; 3 Wildlife Conservation Society Canada, Thunder Bay, Ontario, Canada; 4 Department of Biological Sciences, University of Alberta, Edmonton, Alberta, Canada; 5 Department of Psychology, University of Alberta, Edmonton, Alberta, Canada; 6 Neuroscience and Mental Health Institute, University of Alberta, Edmonton, Alberta, Canada; 7 Department of Evolution, Ecology, and Organismal Biology, The Ohio State University, Columbus, OH, USA; 8 Department of Animal Biology, University of Illinois, Urbana-Champaign, IL, USA; 9 Department of Neurobiology, Physiology and Behavior, University of California Davis, Davis, CA, USA; 10 Department of Mathematics, The Ohio State University, Columbus, OH, USA

**Keywords:** nonapeptide, oxytocin, vasopressin, vasotocin, sociality, cooperative breeding

## Abstract

Social living has evolved numerous times across a diverse array of animal taxa. An open question is how the transition to a social lifestyle has shaped, and been shaped by, the underlying neurohormonal machinery of social behaviour. The nonapeptide neurohormones, implicated in the regulation of social behaviours, are prime candidates for the neuroendocrine substrates of social evolution. Here, we examined the brains of eight cichlid fish species with divergent social systems, comparing the number and size of preoptic neurons that express the nonapeptides isotocin and vasotocin. While controlling for the influence of phylogeny and body size, we found that the highly social cooperatively breeding species (*n* = 4) had fewer parvocellular isotocin neurons than the less social independently breeding species (*n* = 4), suggesting that the evolutionary transition to group living and cooperative breeding was associated with a reduction in the number of these neurons. In a complementary analysis, we found that the size and number of isotocin neurons significantly differentiated the cooperatively breeding from the independently breeding species. Our results suggest that isotocin is related to sociality in cichlids and may provide a mechanistic substrate for the evolution of sociality.

## 1. Introduction

The evolutionary transition from a solitary to a social lifestyle has occurred many times throughout the animal kingdom. An important and open question is whether or not evolution acts on conserved mechanistic pathways (e.g. neural circuits) during these transitions or if there are many possible proximate routes to sociality [1,2]. Across species, the underlying mechanisms regulating social behaviour may be shared and, therefore, we may find similar changes in the neurohormonal machinery controlling the relevant social adaptations among independent social lineages [3]. Thus, it may be possible to detect a consistent mechanistic signature of sociality when comparing highly social species to their less social counterparts. Uncovering such a relationship between sociality and neuronal phenotype would suggest parallelism in the mechanistic basis of social system evolution, helping us to better understand the transition to a social lifestyle.

In order to address this issue, we examined the mechanistic correlates of cooperative breeding as an example of a complex social lifestyle. Cooperative breeding is a social system in which non-breeders belong to social groups and assist in the reproductive efforts of the dominant individuals in their group [4–6]. Cooperative breeders must identify, remember and differentially respond to multiple group members who vary in social status and have distinct individual relationships within the social group [7,8]. Therefore, the transition from independent to cooperative breeding requires behavioural and cognitive adaptations for this heightened level of sociality [9]. For example, cooperative breeders, like other highly social species, must tolerate adult conspecifics other than their mate and offspring [10], and be able to minimize the costs of social conflict [11]. In fishes, cooperative breeding is found only in the lamprologine cichlids of Lake Tanganyika, Africa, where it has arisen several times [12–16]. Multiple closely related cooperative and independently breeding lamprologines live sympatrically, sharing similar diets, biotic and abiotic habitat requirements, and predators [17–20]. Hence these fishes offer an excellent opportunity for comparative analyses of the behavioural and mechanistic underpinnings of complex social lifestyles [14,15,21–23].

In a wide diversity of taxa, the regulation of social behaviour is influenced by the nonapeptides oxytocin and vasopressin (and their non-mammalian homologues), and the impact of these nonapeptides on social behaviour has been well documented [24–28]. In birds and mammals, these nonapeptides have been shown to influence an array of social behaviours and cognitive propensities, including affiliation, bonding, social recognition, social memory, cooperation and aggression [3,29–32]. Many of these behavioural and cognitive characteristics differ between highly social and less social species, including between cooperative and independent breeders [21,33], and thus these nonapeptides provide promising candidates for the proximate substrate of social system evolution [34]. Indeed, nonapeptide circuits have been shown to correlate with social systems in both birds and mammals [10,35–38].

Among teleost fishes, the nonapeptides homologous to oxytocin and vasopressin are known as isotocin and vasotocin, respectively [39]. Extensive evidence has accumulated showing that vasotocin also plays a key role in modulating social behaviour in fishes [40–49]. By contrast, the research on the behavioural role of isotocin is relatively limited [24,50]. However, a small but growing body of work confirms that as with oxytocin in mammals and mesotocin in birds, isotocin is also an important modulator of social behaviour in fishes [22,51–56]. Therefore, both isotocin and vasotocin may be prime proximate targets of social evolution in fishes.

Isotocin and vasotocin are produced in three neuronal groups located in the preoptic area [50], a key brain region for the regulation of social behaviour [34,57]. These areas, known as the parvocellular, magnocellular and gigantocellular populations, can be differentiated by their cell sizes (gigantocellular > magnocellular > parvocellular), by their cytoarchitecture and by their spatial location [58]. Each of these three cell groups projects to the posterior pituitary where nonapeptides are released into the periphery, as well as to diverse targets throughout the brain [59,60], including forebrain regions that have been linked to social behaviour (e.g. the ventral telencephalon [34,57]). Parvocellular, magnocellular and gigantocellular cells appear to serve different functions in the regulation of social behaviour [60,61]. For example, in the African cichlid *Astatotilapia burtoni*, parvocellular vasotocin cells are associated with submissive behaviour in subordinate males while magnocellular cells are associated with aggression in dominant males [45]. Godwin & Thompson [24] hypothesized that this role for magnocellular vasotocin cells in regulating approach and aggression and for parvocellular cells in regulating social withdrawal and submission may be a more general pattern in fishes.

**Figure 1.**
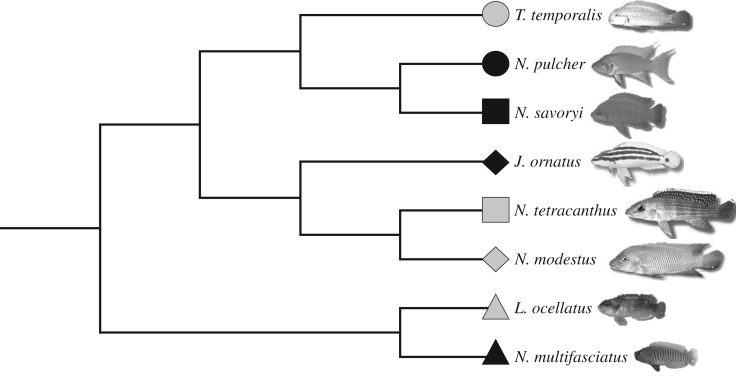
The phylogenetic relationships among the eight species of cichlid fishes included in the current study. Black symbols represent cooperatively breeding species; grey symbols represent independently breeding species. Each shape–colour combination represents a different species.

In this study, we examined the number and size of the parvocellular, magnocellular and gigantocellular isotocin and vasotocin neurons in the preoptic area of each of eight species of lamprologine cichlids using animals collected from the wild (figure 1). We selected four highly social cooperative breeders (*Neolamprologus pulcher*, *N. multifasciatus*, *N. savoryi* and *Julidochromis ornatus*) and four species that are less social independent breeders (*N. tetracanthus*, *N. modestus*, *Telmatochromis temporalis* and *Lamprologus ocellatus*), representing three independent transitions to cooperative breeding [16]. These species live in similar habitats, characterized by a mix of sandy and rocky substrate at depths of 5–15 m, and are exposed to similar environmental conditions in Lake Tanganyika. We compared the number and size of each cell type in each cell group, controlling for body size and phylogenetic relatedness, to look for consistent differences between the cooperatively and independently breeding species. Using a complementary approach, we used discriminant function analyses to determine whether individual fish could be correctly classified into cooperatively or independently breeding social systems based on the size and number of their isotocin or vasotocin cells. A consistent pattern of nonapeptide cell size or number between cooperatively and independently breeding species would suggest that these cell populations were modified in parallel during the emergence of cooperative breeding in the lamprologine cichlids, the only group of fishes to have evolved true cooperative breeding.

## 2. Material and methods

### 2.1. Study site and field methods

All fish were sexually mature males captured from the southern basin of Lake Tanganyika near Mpulungu, Zambia (8°46'52^″^ S, 31°5'18^″^ E) in February–March, 2013. Ten adult males from each of the eight cichlid species were located using SCUBA at depths of 6–12 m and captured using fence- and hand nets. Each fish was slowly brought to the surface, measured for standard body length (the distance from the tip of the snout to the end of the caudal peduncle) with callipers (to 0.1 mm; see the electronic supplementary material, table S1 for the average length of each species), anaesthetized by immersion in a benzocaine solution, and swiftly decapitated. Sex was confirmed by post-mortem examination of the gonads. Whole brains were carefully extracted, and preserved in 4% phosphate-buffered paraformaldehyde prior to transport back to the University of Alberta, Canada.

### 2.2. Histological methods

Prior to immunohistochemistry, brains were cryoprotected in 30% sucrose in 0.1 M phosphate-buffered saline (PBS) for 24 h, embedded in gelatin, and sectioned on the coronal plane at a thickness of 40 µm. Twelve of the 80 brains (six cooperative breeders: two *N. multifasciatus*, two *N. pulcher*, two *N. savoryi*; and six independent breeders: two *L. ocellatus*, three *N. modestus*, one *T. temporalis*) were damaged during extraction from the skull or sectioning and therefore were not used, reducing our final sample size to 68 fish (34 from each social system). Free floating sections were incubated in blocking serum (1 : 10 normal donkey serum, Jackson Immunoresearch Laboratories) with 0.1 M PBS and 0.04% Triton X for 1 h. Tissue was then double-labelled with polyclonal anti-oxytocin (Peninsula Laboratories International; catalogue number T-5021) and anti-vasopressin (Peninsula Laboratories International; catalogue number T-4563) antibodies raised in guinea pigs and rabbits, respectively, against the mammalian forms of oxytocin and vasopressin (1 : 5000, Peninsula Laboratories, San Carlos, CA) with 0.1 M PBS and 10% normal donkey serum for 24 h (−4°C). After rinsing with PBS, immunoreactive isotocin and vasotocin cells were stained by incubating for 2 h in fluorescent secondary antibodies (1 : 200 Alexafluora 594 donkey anti-guinea pig; 1 : 200 Alexafluora 488 donkey anti-rabbit; Jackson Immunoresearch Laboratories; catalogue numbers: 706-005-148 and 711-005-152, respectively). The tissue was then rinsed in PBS again and mounted onto gelatinized slides.

**Figure 2.**
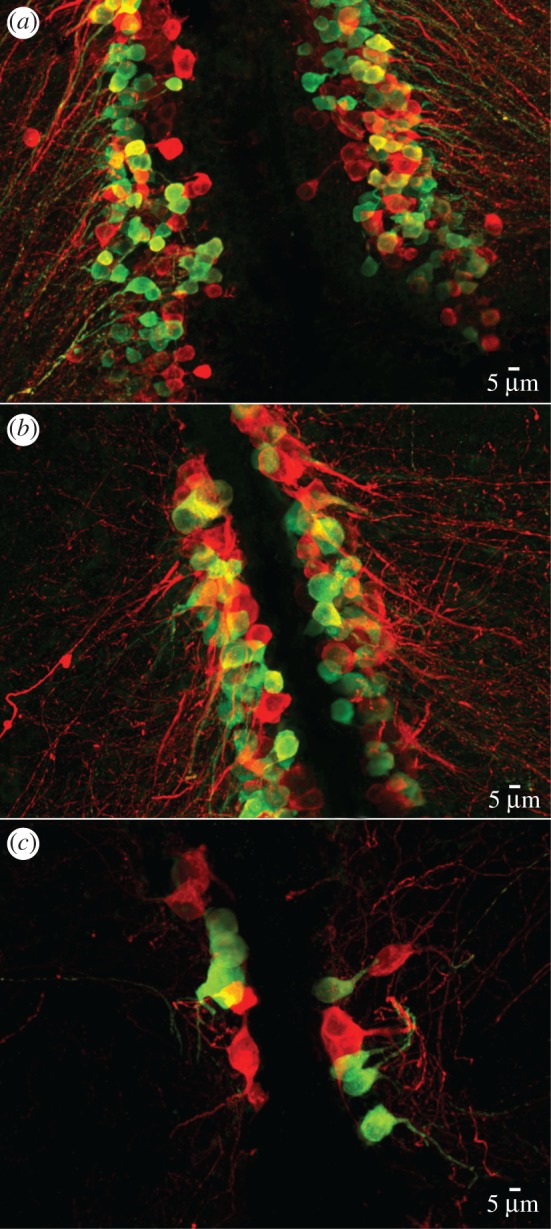
Confocal photomicrographs showing immunohistochemical labelling of nonapeptides in the preoptic area of a wild caught male *Neolamprologus pulcher*. Green cells are vasotocin positive and red cells are isotocin positive. (*a*) Parvocellular, (*b*) magnocellular, (*c*) gigantocellular cell groups. The top of each panel corresponds to the dorsal aspect.

Oxytocin and vasopressin positive neuron cell bodies were visualized with a confocal microscope (Leica TCS SP5) using 488 nm argon ion and 543 nm green HeNe lasers and a 63× water immersion lens. Z-step sizes were adjusted according to the size and density of cell groups in individual brains. The mammalian oxytocin antibody that we used stains both isotocin and vasotocin cells in fishes, while the vasopressin antibody is specific to vasotocin expressing cells (J. Goodson, personal communication). Using a double labelling technique, vasotocin cells were identified as those that were immunoreactive to both the oxytocin and vasopressin antibodies, while the isotocin cells were those stained only by the oxytocin antibody (figure 2). In the fishes studied to date, isotocin and vasotocin cells are intermingled, but each individual cell produces only isotocin or vasotocin (reviewed in [50]). Fiji software (ImageJ version 2.0.0) was used to measure the area of isotocin and vasotocin cells by tracing the circumference of the fluorescently labelled cell body, and cell counts were obtained using the Cell Counter plugin.

Images were scored blind to the social system of each species and the body length of each individual. Vasotocin and isotocin expressing cell bodies were found exclusively in the preoptic area. Each nonapeptide cell group (parvocellular, magnocellular and gigantocellular) was distinguished within each individual fish using a combination of cell size, morphology and location criteria (following [58]; figure 2). We counted all cells of each type in each cell group that showed a clearly discernable perimeter and a visible neurite. We randomly selected cells from which to measure cell body area by assigning a unique integer to each cell and selecting 5% of the cells (minimum 10) of each type, in each group, in each fish using a random number generator.

### 2.3. Phylogenetic tree

Phylogenetic relationships among lamprologine cichlids are complicated by introgressive hybridization, which makes phylogenies constructed solely from mitochondrial DNA unreliable for some species [62–64]. Therefore, we used a recent tree for the lamprologines [16] that estimates the phylogenetic relationships for 69 species of lamprologine cichlids based on three mitochondrial and six nuclear nucleotide sequences, using a Bayesian Markov chain Monte Carlo model. For the purposes of the current study, the consensus tree from Dey *et al*. [16] was trimmed to include only the eight species of interest and visualized using MESQUITE v. 3.10 [65] (figure 1).

### 2.4. Statistical analyses

We used Bayesian phylogenetically controlled statistical analyses to test for associations between social system and isotocin cell count, isotocin cell area, vasotocin cell count and vasotocin cell area. We included cell group (parvocellular, magnocellular and gigantocellular), along with fish identity as a random effect. Because body size has been shown to correlate with nonapeptide cell size and number in other fish species [61,66], we also included body length as a covariate. When our model revealed a significant effect of cell group, we conducted post-hoc tests of the association between social system and nonapeptide cell count or cell area separately for each cell group, including body length as a covariate, and fish identity as a random effect.

For all models and post-hoc tests, we used the package ‘MCMCglmm’ [67] to perform generalized linear mixed models based on a Markov chain Monte Carlo algorithm. Within the MCMCglmm package, the phylogenetically controlled analysis is implemented by including the phylogenetic tree as a random factor in the model (ç). Following examples from de Villemereuil & Nakagawa [68], we defined our priors for the model as *V* = 1 and *ν* = 0.02 for both random effects and the residual variance, which correspond to an inverse-Gamma distribution with shape and scale parameters equal to 0.01, which is canonical [69]. We ran each model for 5 million iterations, with a burnin of 1000, and a thinning interval of 500. With these priors and settings, there was no autocorrelation between successive stored iterations for any of the models [70]. Because Bayesian statistics are based on iterative processes, the outcomes can vary slightly between runs. Therefore, we repeated the analyses three times, and report mean values for the 95% highest posterior density interval (HPD), as well as the *P*_MCMC_, which are the Bayesian equivalents of 95% confidence intervals and *p*-values, respectively. Associations were considered significant when the 95% HPD excluded zero, and *P*_MCMC_ was less than 0.05. The Bayesian phylogenetically controlled analyses were conducted using R v. 3.2.1 within R Studio.

In order to further examine whether the isotocin or vasotocin neuronal phenotypes differed predictably between cooperatively and independently breeding species, we conducted a discriminant function analysis for each nonapeptide. We included this supplementary analysis because discriminant function analysis is a sensitive method for studying group differences among several variables simultaneously [71]. To control for body size, we used the residuals of the linear regression of body length on each variable in our analyses. To test for the predictive ability of the resultant discriminant functions, we used a leave-one-out cross-validation process, wherein each animal is classified based on the discriminant function computed while excluding that individual, resulting in a conservative test of predictive power [72]. The discriminant function analyses were done using IBM SPSS Statistics version 23.

## 3. Results

### 3.1. Phylogenetically controlled analyses

**Figure 3.**
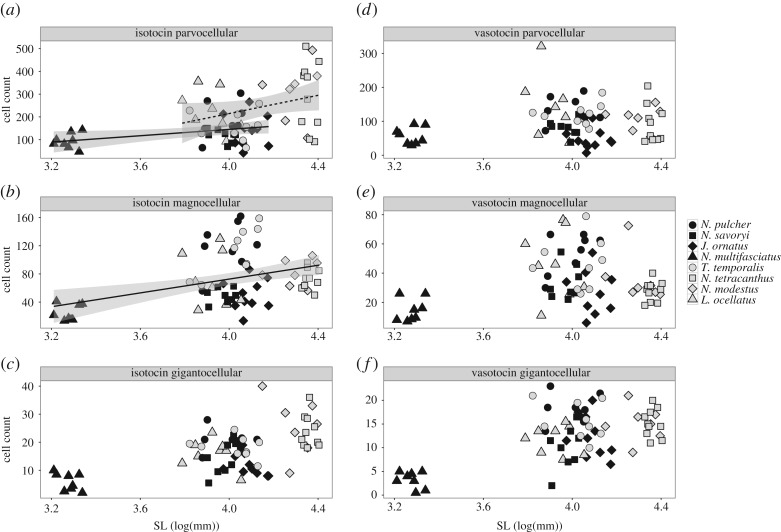
Isotocin (*a*–*c*) and vasotocin (*d*–*f*) cell counts plotted against standard length (SL) for each of eight species of lamprologine cichlid fishes. Black symbols represent cooperatively breeding species; grey symbols represent independently breeding species. Fit lines indicate a significant relationship between body length and cell count (*P*_MCMC_ < 0.05) while separate fit lines for cooperatively (solid line) and independently (dashed line) breeding species indicate a social system difference in cell count (*P*_MCMC_ < 0.05).

**Figure 4.**
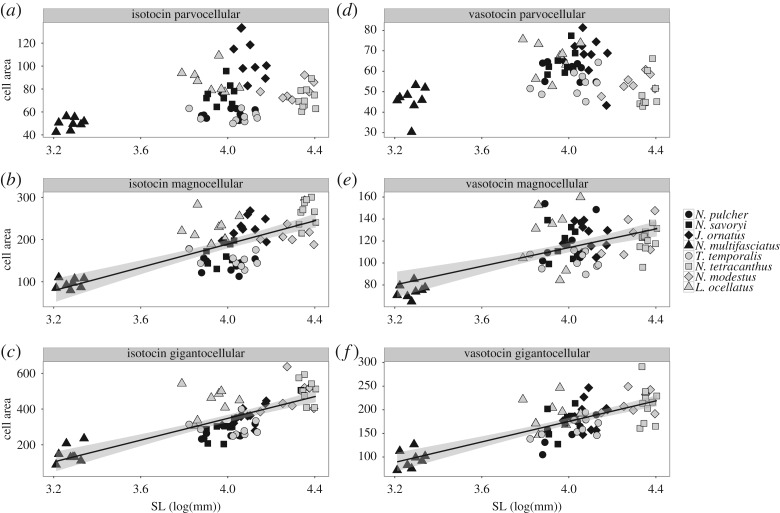
Average isotocin (*a*–*c*) and vasotocin (*d*–*f*) cell areas plotted against standard length (SL) for each of eight species of lamprologine cichlid fishes. Black symbols represent cooperatively breeding species; grey symbols represent independently breeding species. Fit lines indicate a significant relationship between standard length and cell area (*P*_MCMC_ < 0.05).

Cooperatively breeding species had fewer isotocin cells in their preoptic area than the independently breeding species (table 1). More specifically, the cooperative breeders had fewer parvocellular isotocin cells than the independent breeders (table 2 and figure 3*a*). There was no association between magnocellular or gigantocellular isotocin cell counts and social system (table 2 and figure 3*b,c*). We did not detect any relationship between social system and vasotocin cell count (table 1 and figure 3*d–f*). Also there was no relationship between social system and isotocin (table 1 and figure 4*a–c*) or vasotocin cell area (table 1 and figure 4*d–f*).

**Table 1.** Results of Bayesian phylogenetically controlled generalized linear mixed models to test for associations between social system and: isotocin cell count, isotocin cell area, vasotocin cell count and vasotocin cell area. We included cell group (parvocellular, magnocellular and gigantocellular) and body length as covariates. For cell group, magnocellular and gigantocellular were assessed relative to parvocellular. Fish identity was included as a random effect. Because Bayesian statistics are based on iterative processes, the outcomes can vary slightly between runs. Therefore, we repeated the analyses three times, and report mean values for the 95% highest posterior density interval (HPD), as well as the *P*_MCMC_, which are the Bayesian equivalents of 95% confidence intervals and *p*-values, respectively. Fixed effects in italics are considered statistically significant (i.e. the 95% HPD excludes zero, and *P*_MCMC_ is less than 0.05). For full statistical details, see Material and methods.

independent variable	fixed effects	95% HPD		*P*_MCMC_
isotocin cell count	*social system*	*2*.*7*	*62*.*4*	*0*.*03*
	body size (SL)	2.6	99.4	0.07
	*brain area* (*magnocellular*)	*36*.*1*	*77*.*7*	<*0*.*001*
	*brain region* (*gigantocellular*)	*151*.*9*	*192*.*8*	<*0*.*001*
vasotocin cell count	social system	−8.4	48.1	0.12
	body size (SL)	−41.3	42.4	0.77
	*brain area* (*magnocellular*)	*12*.*3*	*33*.*6*	<*0*.*001*
	*brain region* (*gigantocellular*)	*72*.*4*	*92*.*5*	<*0*.*001*
isotocin cell area	social system	−29.4	92.2	0.24
	*body size* (*SL*)	*44*.*1*	*223*.*1*	*0*.*008*
	*brain area* (*magnocellular*)	−*180*.*2*	−*140*.*5*	<*0*.*001*
	*brain region* (*gigantocellular*)	−*294*.*5*	−*254*.*4*	<*0*.*001*
vasotocin cell area	social system	−21.1	16.4	0.79
	*body size* (*SL*)	*33*.*7*	*93*.*5*	<*0*.*001*
	*brain area* (*magnocellular*)	−*68*.*7*	−*53*.*3*	<*0*.*001*
	*brain region* (*gigantocellular*)	−*126*.*1*	−*109*.*6*	<*0*.*001*

**Table 2:** All of our initial models revealed a significant effect of cell group (see the electronic supplementary material, table S1). Therefore, we conducted post-hoc tests of the association between social system and nonapeptide cell count or cell area separately for each cell group. As above, these results were generated using Bayesian phylogenetically controlled generalized linear mixed models, including body length as a covariate, and fish identity as a random effect. Mean values for the 95% highest posterior density interval (HPD) and *P*_MCMC_ are presented. Fixed effects in italics are considered statistically significant (i.e. the 95% HPD excludes zero, and *P*_MCMC_ is less than 0.05). For full statistical details, see Material and methods.

independent variable		fixed effects	95% HPD		*P*_MCMC_
isotocin cell count	parvocellular	*social system*	*5*.*8*	*143*.*4*	*0*.*03*
		body size (SL)	−21.4	236.2	0.13
	magnocellular	social system	−51.2	54.7	0.78
		*body size* (*SL*)	*10*.*9*	*147*.*7*	*0*.*03*
	gigantocellular	social system	−1.0	14.0	0.06
		body size (SL)	−9.9	18.5	0.55
vasotocin cell count	parvocellular	social system	−11.9	106.9	0.10
		body size (SL)	−105.7	85.6	0.93
	magnocellular	social system	−18.5	30.6	0.52
		body size (SL)	−21.5	47.5	0.32
	gigantocellular	social system	−2.3	11.3	0.28
		body size (SL)	−7.9	12.9	0.56
isotocin cell area	parvocellular	social system	−30.4	35.1	0.96
		body size (SL)	−19.0	50.9	0.34
	magnocellular	social system	−32.4	112.4	0.20
		body size (SL)	−7.7	146.2	0.08
	gigantocellular	social system	−14.4	164.2	0.07
		*body size* (*SL*)	*106*.*3*	*386*.*2*	<*0*.*001*
vasotocin cell area	parvocellular	social system	−18.6	10.4	0.62
		body size (SL)	−5.9	34.2	0.10
	magnocellular	social system	−27.0	10.3	0.38
		*body size* (*SL*)	*24*.*9*	*84*.*4*	*0*.*004*
	gigantocellular	social system	−18.1	31.6	0.56
		*body size* (*SL*)	*72*.*8*	*159*.*0*	<*0*.*001*

### 3.2. Discriminant function analyses

**Figure 5.**
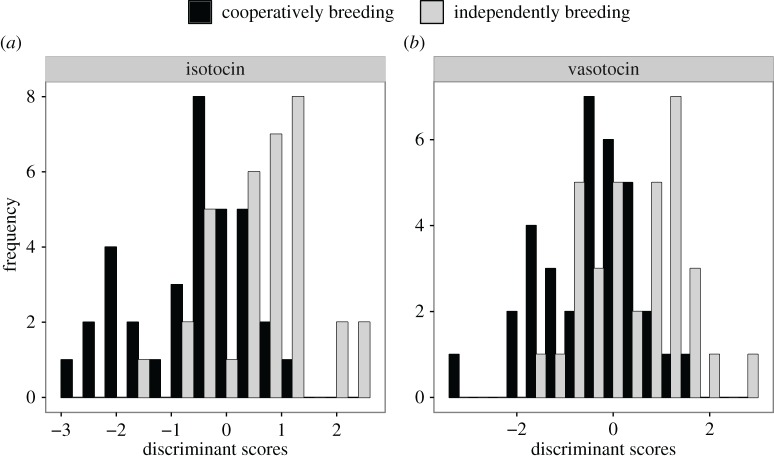
Discriminant function scores for (*a*) isotocin neuronal phenotypes and (*b*) vasotocin neuronal phenotypes. Individual fish were significantly classified into their cross-validated social system using their isotocin (*p* < 0.05) but not their vasotocin neuronal phenotype (*p* > 0.05).

Using both the number and size of the isotocin neurons in each of the parvocellular, magnocellular and gigantocellular areas as predictors, the discriminant function analysis was able to correctly classify individuals into cooperatively or independently breeding systems (Wilks *λ* = 0.71, *χ*^2^ = 21.49, d.f. = 6, *p* = 0.001; figure 5*a*). The isotocin analysis correctly classified 72% of individuals into cross-validated social systems. The size and number of vasotocin cells; however, did not result in the accurate classification of fish into social system (Wilks *λ* = 0.83, *χ*^2^ = 11.48, d.f. = 6, *p* = 0.08; figure 5*b*), correctly classifying only 57% of individuals as cooperative or independent breeders.

## 4. Discussion

We detected an association between the number of isotocin expressing cells in the preoptic area and social system in cichlid fishes. Specifically, in our sample of eight species of lamprologine cichlids, all collected in the wild, we found that males from highly social cooperatively breeding species had fewer parvocellular isotocin cells in their preoptic area than did males from less social independently breeding species, even after accounting for body size and phylogenetic relatedness. Furthermore, the fishes in our sample could be classified successfully into their social system on the basis of their isotocin neurons alone, suggesting that isotocin neuronal phenotypes differ systematically between cooperatively and independently breeding lamprologine cichlids. We did not detect any such differences between cooperative and independent breeders in vasotocin cell size or number, and fish could not be classified into their social system based on their vasotocin cells.

In this study, we have shown for the first time that isotocin neuronal phenotypes differ among closely related species of fishes and that these differences in neurons map on to the variation observed in social systems. Previous work in one of our sampled species, the cooperatively breeding *N. pulcher*, suggests that isotocin regulates social behaviour. For example, exogenous administrations of isotocin reduced shoaling motivation among unfamiliar individuals, while blocking isotocin had the opposite effect [53]. *Neolamprologus pulcher* with higher levels of free isotocin in their brains showed lower rates of affiliative behaviour than did individuals with lower levels of this peptide [54]. Collectively, these results suggest that isotocin may inhibit affiliative tendency or social tolerance. In mammals, oxytocin is typically characterized as stimulating pro-social behaviour; however, oxytocin may also reduce social tendencies and promote anti-social behaviours in some species and/or social contexts [73]. Because isotocin is likely to have different functions depending on where in the brain it is released and with which receptors it interacts [27], more information about the precise role of each of the three isotocin cell populations, their projections throughout the brain and their patterns of receptor binding will be essential to unravel the complete role of isotocin in modulating social behaviour in the lamprologines. Our current data suggest that the parvocellular region is potentially important in generating differences in social behaviour among closely related cichlid species. Future work should endeavour to examine the effects of isotocin release on different parvocellular targets and to correlate parvocellular isotocin levels with observations of social behaviour within individuals.

Previous molecular studies have found that cooperatively breeding and independently breeding lamprologine cichlids do not show a consistent pattern of isotocin brain gene expression, with some cooperatively breeding species showing higher expression of isotocin than their independently breeding relatives while other cooperative breeders show lower expression or no difference [14,22]. There are several possible reasons why our isotocin cell count data contrast with the previous data on isotocin brain gene expression. First, measures of whole brain gene expression capture isotocin transcription occurring in all three cell groups, and thus could have obscured the pattern we observed in the parvocellular region. However, it is worth noting that the parvocellular difference that we observed was strong enough to drive an overall difference in isotocin cell number across cell populations. Second, cell size or number data may contrast with gene expression data (e.g. [74]) and higher isotocin gene expression could indicate greater production of isotocin, while a greater number or larger size of isotocin cells may indicate greater storage [75]. It is possible that cooperative breeders store less of the peptide and instead turn it over more rapidly, through either central signalling or release into the periphery [48]. For example, dominant *N. pulcher* have higher vasotocin gene expression in their brains than do subordinate group members [76], but subordinates have higher levels of free vasotocin in their brains [54], suggesting a possible production versus storage discrepancy. Our study highlights the need for multiple complementary approaches in order to understand the role of the nonapeptides in regulating social behaviour within and across species.

Social and mating system differences between species of birds and mammals tend to be mediated by differences in the number and location of nonapeptide receptors in the brain rather than differences in the nonapeptide producing cells themselves, which tend to be highly consistent in the species studied thus far [10,73,77–79]. Mammals in particular show dramatic species differences in nonapeptide receptor distribution; however, the links to sociality vary in direction and magnitude across species [73]. The current data on nonapeptide receptors in fish brains is limited, particularly for isotocin [24,50,60,80], and this would be a fertile area for future work. A key implication of our results is that, in contrast to mammals, nonapeptide production or storage in the preoptic area can differ among related species in relation to their social behaviour.

We did not find any consistent association between cooperative breeding and the vasotocin neurons in our sample of eight lamprologine cichlid fishes. Our results contrast with Dewan *et al*. [81], who found that a shoaling species of butterfly fish (*Chaetodon miliaris*) had larger vasotocin cells in their preoptic area when compared with a closely related solitary species (*Chaetodon multicinctus*). Although it is not possible to conclusively attribute neural differences to differences in social system by comparing only a single pair of species, their findings do suggest that vasotocin neurons can differ between closely related fish species that differ in social system. Our null result with respect to vasotocin suggests that vasotocin neurons are less consistently associated with social system in the lamprologine cichlids than are isotocin neurons, although further work on individuals of both sexes from a greater variety of species coupled with data on social status and individual behaviour will be required to fully understand the relationship between social system and vasotocin across cichlids.

The cause and effect relationship between neuronal phenotype and social system is not necessarily straightforward. Because social context can affect nonapeptide neuronal phenotypes (e.g. [82]), differences between cooperative and independent breeders may be a consequence rather than a cause of the different social organizations that these fishes live within. Early life experience in a social group could also have organizational effects on nonapeptide neuronal phenotypes and therefore developmental conditions rather than evolved diversity may partially explain distinctions between cooperatively and independently breeding lamprologines in their isotocin neuronal phenotype. Controlled developmental experiments in the laboratory (e.g. [83–85]) will be required to disentangle these possibilities and conclusively rule out plastic differences in nonapeptide cells between cooperative and independent breeders. The species that we examined here would be amenable to such controlled experimentation.

We found that cooperatively breeding lamprologine cichlids differ from their closest independently breeding relatives in isotocin but not vasotocin neuronal phenotypes. Controlling for phylogeny and body size, cooperative breeders had fewer parvocellular isotocin cells than did the independent breeders. Future studies should aim to examine isotocin circuits in more detail, and in particular a comparison of receptor distributions between cooperatively and independently breeding species would be valuable. Additionally, controlled laboratory experiments aimed at mapping individual variation in social behaviour onto nonapeptide producing cells or establishing the effects of developmental environment and social context on nonapeptide neuronal phenotype in these cichlids would likely be illuminating. Future work should also examine both males and females, as sex differences in nonapeptide circuits and function have been observed in other fish species (e.g. [61,66,74,86]). Our study highlights isotocin as a potential mechanistic substrate of social system evolution in the most speciose group of vertebrates, the teleost fishes.

## Supplementary Material

Nonapeptide neuronal phenotypes in eight species of lamprologine cichlid fishes

## Supplementary Material

Average body sizes for eight species of lamprologine cichlid fishes

## Data accessibility

The full dataset is available as the electronic supplementary material.
