# Supplementary material for: Isotocin neuronal phenotypes differ among social systems in cichlid fishes
Source: R Soc Open Sci. 2017 May 17;4(5):170350. doi: 10.1098/rsos.170350 (PMC5451842; doi:10.1098/rsos.170350)
Supplement: Average body sizes for eight species of lamprologine cichlid fishes [file rsos170350supp2.docx]

*Supplementary Table 1.* Mean (±S.E.M.) standard length for each of the 8 studied species.

| Species | Standard length (mm) | Social system |
| --- | --- | --- |
| *Julidochromis ornatus*  *Neolamprologus multifasciatus*  *Neolamprologus pulcher*  *Neolamprologus savoryi* | 59.63±1.18  26.36±0.39  54.32±1.40  52.81±0.90 | Cooperative breeder  Cooperative breeder  Cooperative breeder  Cooperative breeder |
| *Lamprologus ocellatus*  *Neolamprologus modestus*  *Neolamprologus tetracanthus*  *Telmatochromis temporalis* | 51.17±1.29  74.27±1.73  78.28±0.67  54.81±2.31 | Independent breeder  Independent breeder Independent breeder  Independent breeder |
